# Enhancer Control of MicroRNA miR-155 Expression in Epstein-Barr Virus-Infected B Cells

**DOI:** 10.1128/JVI.00716-18

**Published:** 2018-09-12

**Authors:** C. David Wood, Thomas Carvell, Andrea Gunnell, Opeoluwa O. Ojeniyi, Cameron Osborne, Michelle J. West

**Affiliations:** a School of Life Sciences, University of Sussex, Falmer, Brighton, United Kingdom; b Department of Medical and Molecular Genetics, King's College London School of Medicine, Guy's Hospital, London, United Kingdom; Northwestern University

**Keywords:** EBNA2, Epstein-Barr virus, enhancer, miR-155, transcriptional regulation

## Abstract

The oncogenic microRNA (miRNA) miR-155 is the most frequently upregulated miRNA in Epstein-Barr virus (EBV)-positive B cell malignancies and is upregulated in other nonviral lymphomas. Both EBV nuclear antigen 2 (EBNA2) and the B cell transcription factor interferon regulatory factor 4 (IRF4) are known to activate transcription of the host cell gene from which miR-155 is processed (*miR-155HG*; BIC). EBNA2 also activates *IRF4* transcription, indicating that EBV may upregulate miR-155 through direct and indirect mechanisms. The mechanism of transcriptional regulation of *IRF4* and *miR-155HG* by EBNA2, however, has not been defined. We demonstrate that EBNA2 can activate *IRF4* and *miR-155HG* expression through specific upstream enhancers that are dependent on the Notch signaling transcription factor RBPJ, a known binding partner of EBNA2. We demonstrate that in addition to the activation of the *miR-155HG* promoter, IRF4 can also activate *miR-155HG* via the upstream enhancer also targeted by EBNA2. Gene editing to remove the EBNA2- and IRF4-responsive *miR-155HG* enhancer located 60 kb upstream of *miR-155HG* led to reduced *miR-155HG* expression in EBV-infected cells. Our data therefore demonstrate that specific RBPJ-dependent enhancers regulate the IRF4–miR-155 expression network and play a key role in the maintenance of miR-155 expression in EBV-infected B cells. These findings provide important insights that will improve our understanding of miR-155 control in B cell malignancies.

**IMPORTANCE** MicroRNA miR-155 is expressed at high levels in many human cancers, particularly lymphomas. Epstein-Barr virus (EBV) infects human B cells and drives the development of numerous lymphomas. Two genes carried by EBV (LMP1 and EBNA2) upregulate miR-155 expression, and miR-155 expression is required for the growth of EBV-infected B cells. We show that the EBV transcription factor EBNA2 upregulates miR-155 expression by activating an enhancer upstream from the miR-155 host gene (*miR-155HG*) from which miR-155 is derived. We show that EBNA2 also indirectly activates *miR-155* expression through enhancer-mediated activation of *IRF4*. IRF4 then activates both the *miR-155HG* promoter and the upstream enhancer, independently of EBNA2. Gene editing to remove the *miR-155HG* enhancer leads to a reduction in *miR-155HG* expression. We therefore identify enhancer-mediated activation of *miR-155HG* as a critical step in promoting B cell growth and a likely contributor to lymphoma development.

## Abstract (precis)

MicroRNA miR-155 is expressed at high levels in many human cancers, particularly lymphomas. Epstein-Barr virus (EBV) infects human B cells and drives the development of numerous lymphomas. Two genes carried by EBV (LMP1 and EBNA2) upregulate miR-155 expression, and miR-155 expression is required for the growth of EBV-infected B cells. We show that the EBV transcription factor EBNA2 upregulates miR-155 expression by activating an enhancer upstream from the miR-155 host gene (*miR-155HG*) from which miR-155 is derived. We show that EBNA2 also indirectly activates *miR-155* expression through enhancer-mediated activation of *IRF4*. IRF4 then activates both the *miR-155HG* promoter and the upstream enhancer, independently of EBNA2. Gene editing to remove the *miR-155HG* enhancer leads to a reduction in *miR-155HG* expression. We therefore identify enhancer-mediated activation of *miR-155HG* as a critical step in promoting B cell growth and a likely contributor to lymphoma development.

## INTRODUCTION

The oncogenic microRNA (miRNA) miR-155 maps within and is processed from a noncoding RNA transcribed from the B cell integration cluster (*BIC*) gene by RNA polymerase II (Pol II) (1). *BIC* was previously identified as a proto-oncogene activated by proviral insertion in avian leucosis virus-induced lymphomas (2, 3). The miR-155 locus is highly conserved across species, and in humans, it lies within the third exon of *BIC* (miR-155 host gene [*miR-155HG*]). miR-155 appears to play a key role in the regulation of B lymphocyte function. Transcription of *miR-155HG* is activated upon B cell receptor signaling, and in murine models, dysfunction or loss of miR-155 in B lymphocytes causes a severe decrease in antibody-induced signaling (4, 5). Overexpression of miR-155 in mice results in the development of precursor B lymphoproliferative disorders and B cell lymphomas (6). miR-155 expression is highly upregulated in a number of human lymphomas, including Hodgkin's lymphoma (HL) and diffuse large cell B cell lymphoma (DLBCL) (4, 7, 8). The basis of the oncogenic activity of miR-155 has not been fully elucidated; however, a number of target genes that regulate B cell proliferation and survival have been identified. These include transcription regulators, receptors, and signaling pathway components, e.g., *HDAC4*, *PIK3R1*, *SMAD5*, *SHIP1*, *PU.1*, *BCL2*, and *C/EBP*β (9, 10).

Epstein-Barr virus (EBV) is a human herpesvirus that immortalizes B lymphocytes and is associated with the development of numerous lymphomas, including Burkitt's lymphoma (BL), HL, and DLBCL. miR-155 expression is upregulated upon B cell infection by EBV (11). In *in vitro* EBV-transformed B cell lines (lymphoblastoid cell lines [LCLs]) and in an EBV-positive DLBCL cell line, loss of miR-155 expression inhibits cell growth and induces apoptosis, indicating that miR-155 expression is important for transformed B cell survival (12). miR-155 expression in LCLs appears to attenuate high levels of NF-κB signaling, and this may help promote B cell proliferation and prevent apoptosis (13). Consistent with a key role for gene regulation by miR-155 in virus-induced oncogenesis, the oncogenic herpesviruses Kaposi's sarcoma herpesvirus and Marek's disease herpesvirus harbor miR-155 mimics in their viral genomes (14–16).

Two EBV genes essential for B cell transformation upregulate miR-155 expression: the constitutively active CD40 receptor mimic latent membrane protein 1 (LMP1) and the viral transcription factor (TF) Epstein-Barr virus nuclear antigen 2 (EBNA2) (12, 13). The expression of either LMP1 or EBNA2 independently activates transcription of *miR-155HG* (13). Upregulation of AP-1 and NF-κB activity by LMP1 appears to play an important role in the activation of the miR-155 promoter in EBV-infected cells (17, 18). The mechanism of EBNA2 activation of miR-155 has not been demonstrated. EBNA2 is required for B cell immortalization by EBV and activates all viral gene promoters, including LMP1, so indirect activation of miR-155 via upregulation of LMP1 is a likely consequence of EBNA2 expression (19, 20). However, EBNA2 also deregulates host gene transcription by binding to promoter and enhancer elements (21, 22). Enhancer and super-enhancer activation by EBNA2 appears to be widespread in the B cell genome (22–24). For example, EBNA2 activation of the *MYC* proto-oncogene is directed by the targeting of upstream enhancers and modulation of enhancer-promoter looping (21, 25). EBNA2 does not bind DNA directly and associates with viral and cellular gene regulatory elements through its interactions with cellular transcription factors that include RBPJ, PU.1, and EBF1 (26).

An EBNA2-bound super-enhancer postulated to control miR-155 expression was identified in LCLs based on the binding of a number of EBV transcription factors (EBNA2, EBNA3A, EBNA3C, and EBNA-LP), the binding of NF-κB subunits, and broad and high-level histone H3 lysine 27 acetylation (H3K27ac) signals (24). However, the original region identified actually comprises the highly expressed 20-kb *miR-155HG* transcription unit from which miR-155 is derived. A subsequent study using RNA Pol II chromatin interaction analysis by paired-end tag sequencing (ChIA-PET) found that RNA Pol II associated with a number of EBNA2-bound promoter, enhancer, and super-enhancer regions upstream of *miR-155HG* that formed links with the *miR-155HG* promoter (27). However, whether EBNA2 can activate *miR-155HG* transcription via the *miR-155HG* promoter or these putative enhancer elements has not been investigated.

miR-155 expression is also activated by interferon regulatory factor 4 (IRF4) through an interferon-stimulated response element (ISRE) in the *miR-155HG* promoter (28). Interestingly, IRF4 levels are highly upregulated in EBV-infected cells, and like miR-155, *IRF4* is also induced by both LMP1 and EBNA2 (29). As a result, *IRF4* and miR-155 levels correlate in EBV-infected cells. In addition to the potential indirect effects of EBNA2 on *IRF4* expression via LMP1 upregulation, conditional expression of EBNA2 in the presence of protein synthesis inhibitors also demonstrates that *IRF4* is a direct target gene of EBNA2 (30). The mechanism of EBNA2 activation of *IRF4* has not been demonstrated. IRF4 expression is essential for the growth and survival of LCLs, and apoptosis induced by IRF4 depletion can be partially rescued by the expression of miR-155 (28, 31). This indicates that the upregulation of miR-155 by IRF4 may be a key component of its essential role in promoting LCL growth.

To obtain information on how the IRF4–miR-155 expression network is controlled by EBV, we investigated the role of putative upstream EBNA2-bound enhancer elements in the regulation of *miR-155HG* and *IRF4* expression. At both gene loci, we identified specific EBNA2-bound enhancer elements that activate the transcription of their respective promoters in an RBPJ-dependent manner. Deletion of the EBNA2-responsive *miR-155HG* enhancer resulted in a decrease in *miR-155HG* transcription in EBV-infected cells, demonstrating its importance for the maintenance of miR-155 expression. These data identify key enhancer elements utilized by EBV for the control of two genes critical for B cell growth, which is relevant to the study of miR-155 and *IRF4* deregulation in other tumor contexts.

(This article was submitted to an online preprint archive [32].)

## RESULTS

### A *miR-155HG* upstream enhancer is activated by EBNA2 through RBPJ.

**FIG 1:**
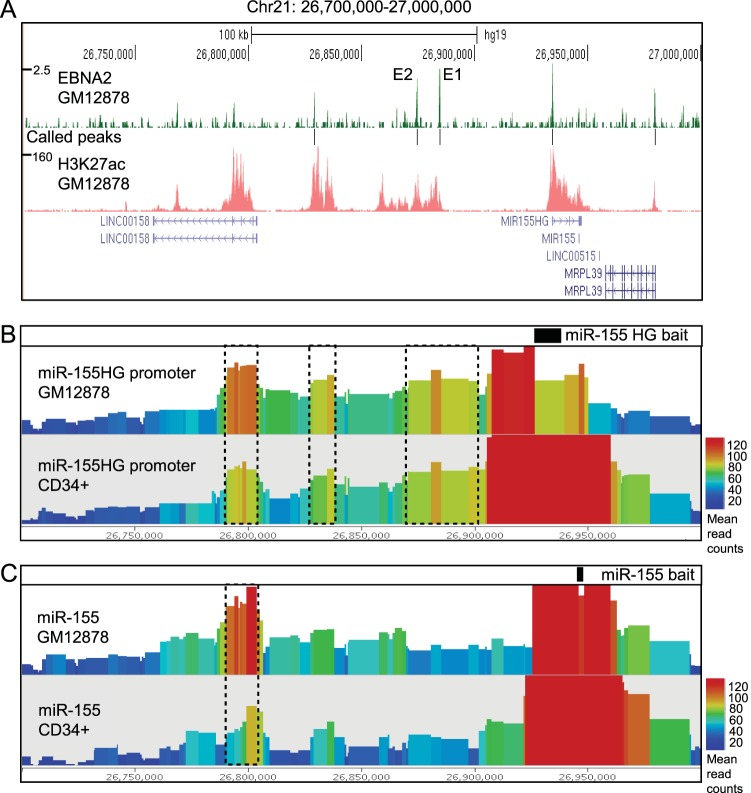
EBNA2 binding and chromosome interactions at the *miR-155HG* locus on chromosome 21. (A) EBNA2 ChIP sequencing in the EBV-infected LCL GM12878 (23) showing the number of sequencing reads from EBNA2-enriched DNA plotted per million input-subtracted total reads and aligned with the human genome. The position of called peaks (model-based analysis of ChIP-seq [MACS], *P* < 10^−7^) is indicated (black lines). The positions of the two main EBNA2-bound putative enhancer regions are indicated (E1 and E2). H3K27ac signals in GM12878 ChIP-seq data available from ENCODE are also shown. (B) Capture Hi-C interaction data obtained using a HindIII fragment encompassing the *miR-155HG* promoter as bait. Interacting fragments were captured from Hi-C libraries generated from GM12878 or CD34^+^ progenitor cells (33). Data show the normalized accumulation of raw read counts of the interaction ditags anchored on the bait regions. The geometric mean shows the average number of reads for each fragment and its immediate neighboring fragments colored according to a rainbow scale. The main interacting regions are shown in boxes with dashed lines. (C) Capture Hi-C interaction data obtained using a HindIII fragment encompassing the miR-155 genomic locus as bait. The main interacting region is shown in the box with dashed lines.

To obtain information on regulatory elements that may control miR-155 expression, we examined *miR-155HG* promoter interaction data obtained from EBV-infected GM12878 LCLs and CD34^+^ hematopoietic progenitor cells using the genome-wide chromosome conformation technique capture Hi-C (CHi-C) (33). In CHi-C, promoter interactions are captured from Hi-C genomic interaction libraries using RNA baits that uniquely hybridize to approximately 22,000 human promoters. These data demonstrate that in both GM12878 and CD34^+^ cells, the *miR-155HG* promoter interacts with three main upstream regions marked by high levels of H3K27ac, indicating transcription regulatory function (Fig. 1A and B). These include two intergenic regions and an intragenic region proximal to the promoter of the *LINC00158* noncoding RNA gene. The same *miR-155HG*-interacting regions were also detected by RNA Pol II ChIA-PET (27). Interestingly, CHi-C data demonstrate that the miR-155 genomic locus within exon 3 of *miR-155HG* interacts with the two intergenic regions at a much lower frequency (Fig. 1C). This suggests that these interactions more frequently involve the *miR-155HG* promoter, consistent with a role in regulating transcription. The miR-155 genomic locus, however, interacts with the *LINC00158* promoter-proximal region, consistent with a gene-to-gene looping interaction between *miR-155HG* and *LINC00158* (Fig. 1C). The *miR-155HG*–*LINC00158* interaction is also the main interaction detected in this region by ChIA-PET for the chromatin-organizing factor CTCF, suggesting that it may be involved in domain organization rather than *miR-155HG* promoter regulation (27). Our EBNA2 chromatin immunoprecipitation sequencing (ChIP-seq) data from the same GM12878 LCL used for CHi-C detect the highest level of EBNA2 binding at two sites within the most proximal intergenic interacting region (23) (Fig. 1A). We therefore investigated the role of these two EBNA2-bound putative enhancers (enhancer 1 [E1] and E2) in the regulation of *miR155-HG*.

**FIG 2:**
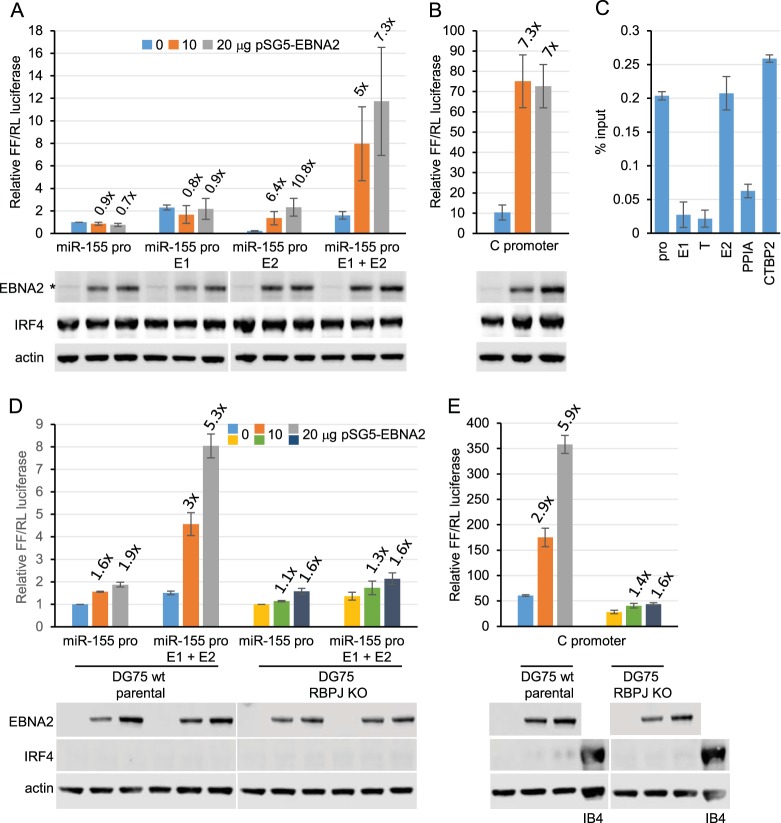
Effects of EBNA2 on *miR-155HG* promoter and enhancer elements. (A) *mIR-155HG* luciferase reporter assays in the presence or absence of EBNA2. DG75 cells were transfected with 2 μg of pGL3 firefly luciferase reporter constructs containing the *miR-155HG* promoter (pro) either alone or in the presence of E1, E2, or both E1 and E2. Assays were carried out in the absence or presence of 10 or 20 μg of the EBNA2-expressing plasmid pSG5-EBNA2 and 0.5 μg of the Renilla luciferase control plasmid (pRL-TK). Firefly (FFL) luciferase signals were normalized to Renilla (RL) luciferase signals and are expressed relative to the signal obtained for the *miR-155HG* promoter in the absence of EBNA2. Results show the means of data from three independent experiments ± standard deviations. Fold activation by EBNA2 relative to the signal obtained for each construct in the absence of EBNA2 is shown above each bar. Western blot analysis of EBNA2 and IRF4 expression is shown below each bar chart, with actin providing a loading control. All blots shown were probed at the same time with the same batch of antibody solution and for each protein show the same exposure. They are therefore directly comparable but have been cut and placed to align with the respective luciferase assay graphs. The asterisk shows the position of a nonspecific band visible upon longer exposures of EBNA2 blots. (B) EBNA2 activation of an EBV C promoter reporter construct was used as a positive control. (C) ChIP-QPCR analysis of RBPJ binding at the *miR-155HG* locus in GM12878 cells. Precipitated DNA was analyzed using primer sets located at the promoter, E1, and E2 and in a trough between E1 and E2 (T). EBNA2 binding at the transcription start site of *PPIA* and at the previously characterized *CTBP2* binding site were used as negative and positive binding controls, respectively. Mean percent input signals, after subtraction of signals for the no-antibody controls, ± standard deviations are shown for three independent ChIP experiments. (D) Luciferase reporter assays carried out using the *miR-155HG* promoter or the *miR-155HG* E1 and E2 constructs in DG75 wt parental cells that lack IRF4 expression and the corresponding RBPJ knockout (KO) cell line. Results are displayed as described above for panel B. (E) Luciferase reporter assays carried out as described above for panel D, using the RBPJ-dependent C promoter reporter construct.

We generated luciferase reporter plasmids containing the *miR-155HG* promoter and one or both enhancer elements. Reporter assays carried out with the EBV-negative B cell line DG75 in the absence or presence of transient EBNA2 expression demonstrated that EBNA2 had no effect on the *miR-155HG* promoter but activated transcription up to approximately 7-fold when a region encompassing both E1 and E2 was inserted upstream of the promoter (Fig. 2A). The level of activation was similar to that observed for the EBNA2-responsive EBV C promoter (Fig. 2B). When testing each enhancer separately, we found that the presence of E1 alone did not convey EBNA2 responsiveness, but it increased basal transcription levels compared to the promoter alone by approximately 2-fold (Fig. 2A). This indicates that E1 has EBNA2-independent enhancer function. EBNA2 activated transcription via E2 alone up to approximately 11-fold, indicating that E2 is an EBNA2-responsive enhancer (Fig. 2A). Interestingly, the presence of E2 decreased basal transcription levels approximately 5-fold compared to the promoter alone (Fig. 2A). This is consistent with the presence of repressive elements in the enhancer that can limit basal transcription activity, a feature that we observed previously for some of the EBNA2-responsive enhancer elements at *RUNX3* and *RUNX1* (23). As a result, the overall level of transcription in the presence of E2 was lower than that in the presence of E1 and E2 combined (Fig. 2A). Since EBNA2 upregulates *IRF4* and IRF4 is a known activator of *miR-155HG*, we investigated whether the effects of EBNA2 in these reporter assays may be indirect and the result of increased endogenous IRF4 expression. We found that transient expression of EBNA2 did not increase endogenous IRF4 expression (Fig. 2A and B). This indicates that longer-term expression or a preformed chromatin signature at the regulatory elements involved is important for the activation of endogenous *IRF4* by EBNA2. We conclude that EBNA2-independent and EBNA2-dependent enhancers regulate *miR-155HG* transcription in EBV-infected cells and that EBNA2 activates transcription directly via association with a specific *miR-155HG* enhancer.

EBNA2 binds to many target gene enhancers through the cell transcription factor RBPJ (CBF1) (21). We investigated whether EBNA2 activation of *miR-155HG* E2 was mediated via RBPJ. ChIP-quantitative PCR (QPCR) analysis of RBPJ binding in the GM12878 LCL detected RBPJ binding at E2 and not E1, consistent with a role for RBPJ in EBNA2 activation of E2 (Fig. 2C). To confirm this, we carried out reporter assays in a DG75 RBPJ knockout cell line (34). This cell line was derived from a different parental DG75 cell line that also lacks IRF4 expression, so for comparison, we also carried out reporter assays in the parental DG75 wild-type (wt) cell line (Fig. 2D). Our data demonstrated that EBNA2 activated the transcription of the *miR-155HG* E1- and E2-containing reporter construct in the wild-type DG75 cell line to the same extent as the EBV C promoter control, confirming our previous results (Fig. 2D). However, in DG75 RBPJ knockout cells, the activation of this reporter construct by EBNA2 was almost completely abolished (Fig. 2D). This mirrored the loss of EBNA2 activation observed for the RBPJ-dependent viral C promoter (Fig. 2E). These data also provide further evidence that EBNA2 activation of *miR-155HG* E2 is not an indirect effect mediated by IRF4 upregulation, and we confirmed that IRF4 expression is not induced by EBNA2 in this cell background (Fig. 2D).

Interestingly, EBNA2 binding sites often coincide with binding sites for IRF4 or IRF4-containing transcription complexes, indicating that IRF4 may be involved in EBNA2 binding to DNA (24, 35). However, our results indicate that IRF4 is not required for EBNA2 targeting of the *miR-155HG* E2 enhancer element since EBNA2 activation was efficient in the absence of IRF4 (Fig. 2D). We conclude that EBNA2 can directly upregulate *miR-155HG* transcription through a distal RBPJ-dependent enhancer (E2) independently of IRF4.

### IRF4 independently activates miR-155HG via promoter and enhancer elements.

**FIG 3:**
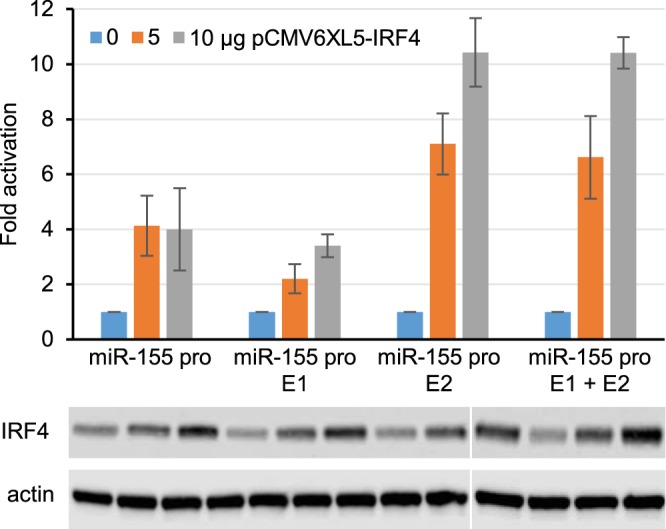
Effects of IRF4 on *miR-155HG* promoter and enhancer elements. DG75 cells were transfected with 2 μg of pGL3 firefly luciferase reporter constructs containing the *miR-155HG* promoter either alone or in the presence of E1, E2, or both E1 and E2. Assays were carried out in the absence or presence of 5 or 10 μg of the IRF4-expressing plasmid pCMV6XL5-IRF4. Data from Western blot analysis of IRF4 and actin expression are shown below the bar chart. Blots shown were probed at the same time with the same batch of antibody solution and for each protein show the same exposure. They are therefore directly comparable but have been cut and placed to align with the respective luciferase assay graphs. One set of samples was split over two gels. Firefly luciferase signals were normalized to actin Western blot signals, and fold activation relative to the signal for each construct in the absence of EBNA2 is shown. Results show the means of data from three independent experiments ± standard deviations.

Our data demonstrate that IRF4 is not required for the effects of EBNA2 on *miR-155HG* transcription. However, IRF4 can independently activate the *miR-155HG* promoter through an ISRE (28). It is not known whether IRF4 can also activate *miR-155HG* transcription through enhancer elements. We therefore tested whether exogenous expression of IRF4 in DG75 cells can activate *miR-155HG* transcription via upstream enhancers. Because IRF4 activates the control plasmid (pRL-TK), firefly reporter activity was normalized to actin expression as a previously described alternative in these assays (36). Consistent with previously reported data, we found that the exogenous expression of IRF4 resulted in a 4-fold increase in *miR-155HG* promoter activity (28). The presence of E1 did not result in any further increase in *miR-155HG* transcription by IRF4 (Fig. 3). However, the additional presence of E2 increased the activation of the *miR-155HG* reporter to approximately 10-fold. These data demonstrate that *miR-155HG* E2 is IRF4 responsive and contributes to IRF4 activation of *miR-155HG* transcription.

Taken together, our results indicate that *miR-155HG* promoter activation by IRF4 and the independent effects of IRF4 and EBNA2 on a specific *miR-155HG* enhancer contribute to the high-level expression of *miR-155HG* and miR-155 in EBV-infected B cells.

### An IRF4 upstream enhancer is activated by EBNA2 through RBPJ.

**FIG 4:**
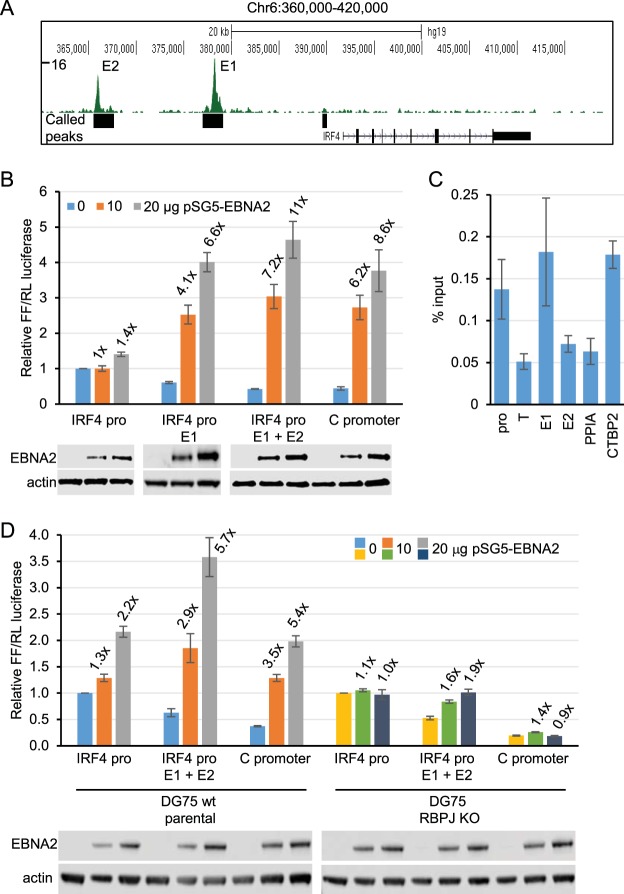
Effects of EBNA2 on *IRF4* promoter and enhancer elements. (A) EBNA2 ChIP sequencing reads at the *IRF4* locus in Mutu III BL cells. The numbers of sequencing reads from EBNA2-enriched DNA are plotted per million input-subtracted total reads and aligned with the human genome. The positions of called peaks (MACS, *P* < 10^−7^) are indicated (black boxes) (22). The positions of the two main EBNA2-bound putative enhancer regions are indicated (E1 and E2). (B) *IRF4* luciferase reporter assays in the presence or absence of EBNA2. DG75 cells were transfected with 2 μg of pGL3 firefly luciferase reporter constructs containing the *IRF4* promoter either alone or in the presence of E1 or both E1 and E2. Assays were carried out in the absence or presence of 10 or 20 μg of the EBNA2-expressing plasmid pSG5-EBNA2 and 0.5 μg of the Renilla luciferase control plasmid (pRL-TK). Firefly luciferase signals were normalized to Renilla luciferase signals and are expressed relative to the signal obtained for the *IRF4* promoter in the absence of EBNA2. EBNA2 activation of an EBV C promoter reporter construct was used as a positive control. Results show the means of data from three independent experiments ± standard deviations. Fold activation by EBNA2 relative to the signal obtained for each construct in the absence of EBNA2 is shown above each bar. Data from Western blot analysis of EBNA2 are shown below each bar chart, with actin providing a loading control. All blots shown were probed at the same time with the same batch of antibody solution and for each protein show the same exposure. They are therefore directly comparable but have been cut and placed to align with the respective luciferase assay graphs. (C) ChIP-QPCR analysis of RBPJ binding at the *IRF4* locus in GM12878 cells. Precipitated DNA was analyzed using primer sets located at the promoter, E1, and E2 and in a trough between the promoter and E1 (T). EBNA2 binding at the transcription start site of *PPIA* and at the previously characterized *CTBP2* binding site were used as negative and positive binding controls, respectively. Mean percent input signals, after subtraction of signals for the no-antibody controls, ± standard deviations are shown for three independent ChIP experiments. (D) Luciferase reporter assays carried out using the *IRF4* promoter, the *IRF4* E1+E2 construct, and the RBPJ-dependent C promoter reporter construct in DG75 wt parental cells and the corresponding RBPJκ knockout cell line. Results are displayed as described above for panel B.

Our data support a role for IRF4 as a key regulator of *miR-155HG* expression in EBV-infected cells. *IRF4* is also an EBNA2 target gene, but the mechanism of *IRF4* upregulation by EBNA2 has not been defined (28, 30). RNA Pol II ChiA-PET analysis recently identified a number of upstream regions that interact with *IRF4* in the GM12878 LCL (27). These regions include the transcription unit of *DUSP22*, an intergenic region upstream from *DUSP22* predicted to be a super-enhancer, and intergenic regions between *IRF4* and *DUSP22*. The upstream super-enhancer was linked to both *DUSP22* and *IRF4* and thus likely represents an important regulatory region (27). EBNA2 ChIP sequencing data that we obtained using EBV-infected cells derived from a BL cell line additionally identified two large EBNA2 binding peaks within the region 35 kb directly upstream of *IRF4* (Fig. 4A). We investigated the potential role of these regions in EBNA2 activation of *IRF4*. These putative proximal and distal EBNA2-bound enhancer regions are referred to as *IRF4* enhancer 1 (E1) and *IRF4* enhancer 2 (E2), respectively (Fig. 4A). Luciferase reporter assays carried out in the two different DG75 cell line clones in the absence or presence of transient EBNA2 expression demonstrated that EBNA2 had little effect on the *IRF4* promoter (Fig. 4B and D). The presence of *IRF4* E1 reduced basal transcription by 2-fold and increased EBNA2 activation up to approximately 7-fold, similar to the level of EBNA2 activation observed for the EBV C promoter (Fig. 4B). The additional inclusion of *IRF4* E2 alongside *IRF4* E1 had little further effect on EBNA2 activation (Fig. 4B). These data indicate that *IRF4* E1 acts as an EBNA2-responsive enhancer. Consistent with EBNA2 activation through RBPJ, ChIP-QPCR detected RBPJ binding at *IRF4* E1 and not E2 (Fig. 4C). Accordingly, EBNA2 activation of the *IRF4* enhancer construct was decreased from approximately 6-fold to approximately 2-fold in RBPJ knockout cells. Our data therefore demonstrate that EBNA2 can activate *IRF4* transcription through an RBPJ-dependent enhancer (E1) located 13 kb upstream from the transcription start site (TSS).

### Deletion of *miR-155HG* E2 from the B cell genome reduces *mIR-155HG* expression.

**FIG 5:**
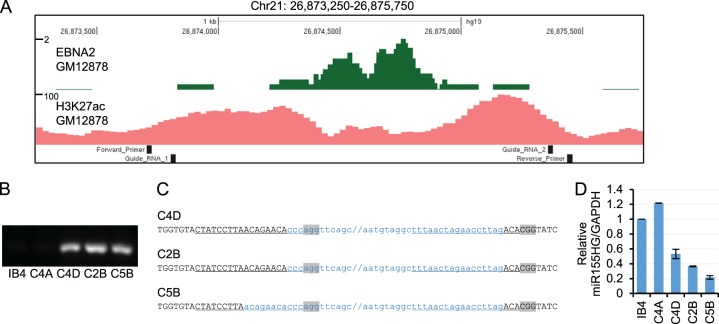
Effects of CRIPSR/Cas9-mediated deletion of *miR-155HG* enhancer 2. (A) EBNA2 ChIP sequencing reads in GM12878 cells (23) and H3K27ac ChIP-seq signals in GM12878 from ENCODE at the *miR-155HG* enhancer 2 region as in Fig. 1. The locations of the guide RNAs used for CRISPR gene editing and the PCR primers used for screening cell clones are indicated. (B) PCR analysis of single-cell clones obtained by limited dilution following transfection of guide RNAs and Cas9 protein using primers that span the deletion site and efficiently amplify a product (180 bp) only from templates carrying an E2 deletion. (C) DNA sequence of the deletion spanning PCR products from the C4A, C2B, and C5B cell lines. Black uppercase type shows the sequence present in the PCR products, and blue lowercase type shows the 5′ and 3′ ends of the deleted region, with forward slashes showing the positions of the remaining ∼1.5 kb of deleted DNA. The sgRNA target sequences are underlined, and PAM sequences are shown in gray. (D) QPCR analysis of total RNA extracted from IB4 parental cells or cell line clones using primers specific for *miR-155HG* and GAPDH. *miR-155HG* signals were normalized by dividing them by GAPDH signals, and expression levels are shown relative to the signal in IB4 parental cells. Results show the means ± standard deviations of data for PCR duplicates from a representative experiment.

Since EBNA2 and IRF4 can activate transcription through *miR-155HG* E2 in reporter assays, we next tested the role of this enhancer in the regulation of *mIR-155HG* in EBV-infected B cells. To do this, we used CRISPR/Cas9 gene editing to remove the region encompassing E2 (Fig. 5A) from the genome of the EBV-immortalized LCL IB4. We designed two single guide RNAs (sgRNAs), one targeting a region 5′ of the enhancer and one targeting a region 3′ of the enhancer, so that DNA repair following Cas9 cleavage would generate an E2 deletion (Fig. 5A). Both sgRNAs comprised 20-nucleotide sequences that target the genomic region adjacent to a protospacer adjacent motif (PAM) required for Cas9 cleavage (Fig. 5C). sgRNAs were transfected into IB4 cells alongside Cas9 protein, and single-cell clones were generated by limiting dilution. PCR screening was used to identify cell line clones containing E2 deletions using a forward primer located 5′ of the E2 region and a reverse primer located 3′ of E2 to amplify a 180-bp DNA product across the deletion site (Fig. 5A and B). This primer set did not amplify DNA from intact templates containing E2, as the amplicon was too large (1.75 kb) for efficient amplification under the conditions used. For three cell line clones tested (C4D, C2B, and C5B), we detected amplification of a 180-bp PCR product consistent with the presence of an E2 deletion (Fig. 5B). We did not detect this PCR product in parental IB4 cells and an additional clone, C4A, indicating that this cell line clone did not contain a deletion (Fig. 5B). Sequencing of the PCR products amplified across the deletion site confirmed the E2 deletion (Fig. 5C). Clones C4D and C2B contained deletions, consistent with cleavage by Cas9 3 bases upstream from the PAM sequence, as expected, and the subsequent ligation of the cleaved ends. Clone C5B had an additional deletion of 8 nucleotides at the 5′ cut site, indicating the loss of a small amount of additional DNA during the DNA repair-and-religation process (Fig. 5C).

We next used real-time PCR analysis to determine whether the deletion of *miR-155HG* E2 affected the levels of endogenous *miR-155HG* RNA in IB4 cells. We found that all three deletion mutant cell line clones had reduced levels of *miR-155HG* transcripts compared to parental IB4 cells or the nondeleted C4A cell line (Fig. 5D). *miR-155HG* RNA expression was reduced by 47%, 63%, and 78% in cell line clones C4D, C2B, and C5B, respectively (Fig. 5D). This indicates that the RBPJ-dependent EBNA2-responsive enhancer (E2) located 60 kb upstream of *miR-155HG* plays an important role in maintaining *miR-155HG* expression in EBV-infected cells. Given that miR-155 is derived by processing of the *miR-155HG* transcript, our data indicate that this enhancer would be important in controlling miR-155 expression.

**FIG 6:**
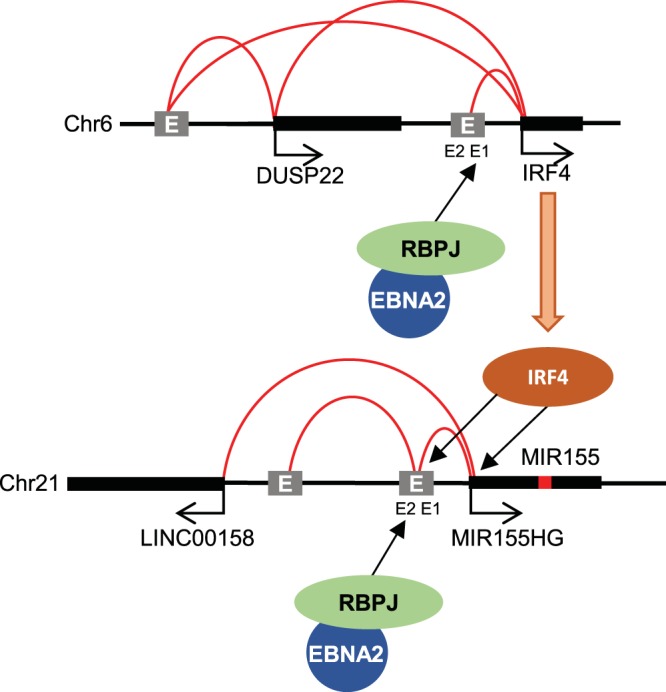
Model for enhancer activation of *IRF4* and *miR-155HG* by EBV EBNA2. EBNA2 targets an intergenic enhancer 13 kb upstream of *IRF4* via RBPJ. A super-enhancer upstream of *DUSP22* bound by EBNA2 also links to both *DUSP22* and *IRF4*. IRF4 then activates *miR-155HG* via the promoter and an intergenic enhancer located 60 kb upstream. EBNA2 activates the *miR-155HG* upstream enhancer via RBPJ. The *miR-155HG* promoter also links to an additional upstream region and the *LINC00158* gene. Curved red lines indicate chromosomal interactions.

In summary, we have identified and characterized new enhancer elements that play a key role in the direct and indirect upregulation of miR-155 expression in EBV-infected cells by the EBV transcription factor EBNA2 (Fig. 6). Importantly, we show that an EBNA2- and IRF4-responsive enhancer element located 60 kb upstream from the *miR-155HG* TSS is essential to maintain high-level *miR-155HG* RNA expression.

## DISCUSSION

### Enhancer control of *miR-155HG* by EBV.

We have characterized an enhancer 60 kb upstream of the miR-155-encoding gene *miR-155HG* that is bound by EBNA2, the key transcriptional regulator encoded by EBV. This enhancer (enhancer 2) was responsive to EBNA2 in reporter assays, and EBNA2 activation was dependent on the expression of the host cell protein RBPJ. Since EBNA2 cannot bind DNA directly, this is in line with EBNA2 binding via its interaction with RBPJ (37, 38). We found that the presence of *miR-155HG* enhancer 2 in the B cell genome was important to maintain the *miR-155HG* expression level in an EBV-infected B cell line. Given that this enhancer is EBNA2 responsive in reporter assays, it is likely that EBNA2 activation of enhancer 2 plays a key role in maintaining *miR-155HG* expression in EBV-infected cells. Since enhancer 2 contains binding sites for a number of other B cell transcription factors (e.g., SPI1 [PU.1], RUNX3, NF-κB RelA, BATF, and SRF), it is possible that these factors also contribute to the maintenance of *miR-155HG* expression in EBV-infected cells. miR-155 depletion has been shown to impair the growth of EBV-infected cells (12), but although we found that deletion of enhancer 2 reduced *miR-155HG* expression, we observed variable growth rates across our control and miR-155 deletion clones (data not shown). It is therefore likely that previous studies using a miRNA sponge to deplete miR-155 achieved much larger reductions in miR-155 levels than we observed. In our cells, miR-155 expression is still detectable as a result of promoter activation by IRF4 and the likely influence of other more distal enhancers (see below), so differences in growth may not be detectable.

miR-155HG enhancer 2 is located within a region upstream of *miR-155HG* that is detected by CHi-C and RNA Pol II ChIA-PET to associate with the *miR-155HG* promoter. Although another putative enhancer bound by EBNA2 in the GM12878 LCL (enhancer 1) is also present in this region, we found that enhancer 1 was not EBNA2 responsive but upregulated transcription from the *miR-155HG* promoter in reporter assays. This indicates that this region possesses EBNA2-independent enhancer function. The detected EBNA2 binding at enhancer 1 may therefore be the consequence of looping between enhancer 1 and enhancer 2 that would lead to the indirect precipitation of this region of DNA in EBNA2 ChIP-seq experiments. This has been described for other TFs (e.g., see reference 39). Interestingly, binding at enhancer 1 is not detected by EBNA2 ChIP-seq in a BL cell background (22), so its activity and looping interactions may be cell type dependent. Indirect immunoprecipitation of the *miR-155HG* promoter as a result of looping interactions with EBNA2-bound enhancers may also explain the presence of an EBNA2 peak at the *miR-155HG* promoter despite the fact that the promoter is not consistently or highly EBNA2 responsive in reporter assays.

Two further upstream regions also interact with the *miR-155HG* promoter by CHi-C and RNA Pol II ChIA-PET in LCLs (one intergenic and one proximal to the *LINC00158* promoter) (Fig. 6). This is consistent with the presence of an active enhancer-promoter hub formed between two intergenic enhancer regions (one of which encompasses enhancer 2) and the promoter-proximal regions of *miR-155HG* and *LINC00158*. In two EBV-infected LCL backgrounds (GM12878 and IB4), maximal EBNA2 (and RBPJ) binding at the *miR-155HG* locus is detected in the intergenic interacting region encompassing *miR-155HG* enhancer 2 (21, 23, 27). This is despite the classification of the remaining intergenic region and the *LINC00158* promoter-proximal region as EBV super-enhancers based on their chromatin and TF landscape profiles (27). It is therefore possible that EBNA2 accesses the *miR-155HG* enhancer hub and upregulates miR-155 expression through its RBPJ-dependent association with *miR-155HG* enhancer 2. Our observations highlight the importance of testing the EBNA2 responsiveness of EBNA2-bound regions rather than relying on binding profiles alone to assign EBNA2 enhancer function.

The constitutively active EBV membrane protein LMP1 also activates *miR-155HG* transcription. NF-κB and AP-1 sites in the *miR-155HG* promoter have been shown to be important to maintain *miR-155HG* promoter activity in LCLs, and two NF-κB sites and the AP-1 site mediate LMP1 responsiveness in transiently transfected EBV-negative cells (17, 18). NF-κB RelA also binds to the *miR-155HG* enhancer 2 region and the putative upstream super-enhancer, so it is also possible that LMP1 activation of the NF-κB and AP-1 pathways also activates *miR-155HG* enhancers. Thus, both promoter (and possibly enhancer) activation by LMP1 and enhancer activation by EBNA2 may contribute to the high-level miR-155 expression observed in EBV-infected cells.

### *miR-155HG* activation by IRF4.

Our results also revealed that the B cell transcription factor IRF4 can activate *miR-155HG* transcription via enhancer 2 in addition to its known effects on the *miR-155HG* promoter. IRF4 activates the *miR-155HG* promoter via an ISRE. There are no ISREs within *miR-155HG* enhancer 2, but the sequence encompassing the PU.1 binding site partially matches an E26 transformation-specific or E-twenty-six (ETS)-IRF composite element (EICE), so IRF4 could bind this element in combination with the ETS protein PU.1. This putative EICE is located approximately 500 bp away from the only match to the RBPJ consensus motif in enhancer 2, so we do not anticipate any competition between IRF4 and EBNA2/RBPJ in the activation of the *miR-155HG* enhancer.

### Enhancer control of *IRF4* by EBV.

EBNA2 also indirectly influences *miR-155HG* expression through the transcriptional upregulation of *IRF4*, and we demonstrated that this is mediated through an upstream enhancer. In addition to the presence of an EBNA2-bound super-enhancer upstream of the neighboring *DUSP22* gene (27), we found that EBNA2 can also upregulate *IRF4* transcription through an RBPJ-dependent enhancer located in an intergenic region 13 kb upstream from *IRF4*. This is consistent with recent reports of *IRF4* as an RBPJ-dependent EBNA2 target gene (40). At *IRF4* and *DUSP22*, EBNA2 therefore likely targets multiple enhancers and super-enhancers.

### miR-155 as a therapeutic target.

miR-155 is overexpressed in many tumor contexts, including hematological malignancies, and is implicated in cancer therapy resistance (10). It therefore represents an important therapeutic target. Preliminary results from a phase I clinical trial using an anti-miRNA to miR-155 in patients with cutaneous T cell lymphoma showed that the anti-miRNA is well tolerated when injected intratumorally (41). Inhibition of miR-155 expression through indirect transcriptional repression has also been tested in acute myeloid leukemia (AML) cells using an inhibitor of the NEDD8-activating enzyme (42). NEDD8-dependent ubiquitin ligases regulate NF-κB activity, and their inhibition by MLN4924 in AML cells results in reduced binding of NF-κB to the *miR-155HG* promoter and a reduction in miR-155 expression. In mice engrafted with leukemic cells, MLN4924 treatment reduced miR-155 expression and increased survival. These data provide evidence for transcriptional inhibition of miR-155 as a therapeutically viable strategy.

The sensitivity of super-enhancers to transcriptional inhibitors is also being exploited as a therapeutic strategy in various tumor contexts. super-enhancers often drive the high-level expression of oncogenes, and super-enhancer inhibition by CDK7 and BET inhibitors can effectively block tumor cell proliferation and enhance survival in mouse models of disease (43–45). miR-155 expression in human umbilical vein endothelial cells is sensitive to inhibition by BET and NF-κB inhibitors (46). This was proposed to result from inhibition of an upstream miR-155 super-enhancer, but the region examined actually represents the *miR-155HG* transcription unit, which has high-level H3K27ac (used as a super-enhancer marker) throughout its length when *miR-155HG* is transcriptionally active. Nonetheless, this study highlights the usefulness of transcription inhibitors in reducing miR-155 expression. Our identification and characterization of the enhancers that drive *miR-155HG* transcription in B cells may therefore underpin therapeutic opportunities for the inhibition of miR-155 expression in numerous B cell cancer contexts where miR-155 is a key driver of tumor cell growth.

## MATERIALS AND METHODS

### Cell lines.

All cell lines were cultured in RPMI 1640 medium (Invitrogen) supplemented with 10% fetal bovine serum (Gibco), 1 U/ml penicillin G, 1 μg/ml streptomycin sulfate, and 292 μg/ml l-glutamine at 37°C in 5% CO_2_. Cells were routinely passaged twice weekly. The DG75 cell line originates from an EBV-negative BL (47). DG75 cells cultured in our laboratory (originally provided by M. Rowe) express low levels of IRF4, but DG75 cells obtained from B. Kempkes (referred to here as DG75 wt parental cells) lack IRF4 expression. The DG75 RBPJ (CBF1) knockout cell line (SM224.9) was derived from DG75 wt parental cells (34). IB4 (48) and GM12878 (obtained from Coriell Cell Repositories) are EBV-immortalized lymphoblastoid cell lines (LCLs) generated by infection of resting B cells *in vitro*.

### Plasmid construction.

The *miR-155HG* promoter sequence from positions −616 to +515 (human GRCh37/hg19 chr21:26933842-26934972) was synthesized by GeneArt Strings (Invitrogen) to include XhoI and HindIII restriction enzyme sites and cloned into pGL3 basic (Promega) to generate the pGL3miR-155HG promoter construct. The pGL3miR-155HG enhancer 1 (E1) construct was generated in a similar way by the synthesis of the promoter and upstream E1 region (chr21:26884583-26885197) as a single DNA fragment that was then cloned into pGL3 basic. To generate the miR-155HG promoter E1+E2 construct, the promoter and E1 and E2 regions (chr21:26873921-26875152) were synthesized as a single DNA fragment and cloned into pGL3 basic. The pGL3miR-155HG promoter E2 construct was generated using sequence- and ligation-independent cloning. The E2 region was amplified by PCR from the miR-155HG promoter E1+E2 construct using primers containing vector and insert sequences (forward primer 5′-TCTTACGCGTGCTAGCCCGGGCTCGAGGAGAGGTTTAAAGCACTCAGACAGC-3′ and reverse primer 5′-GGGCTTTGAGAACGTTTGTACCTCGAGGATCTAGAACCTCTGGAGTTGGAGAT-3′). The pGL3miR-155HG promoter vector was digested with XhoI, and T4 DNA polymerase was then used to further resect the cut ends to allow the insert to anneal to extended single-stranded regions of the vector. Single-strand DNA gap filling occurred through DNA repair following transformation of the plasmid into Escherichia coli.

The *IRF4* promoter sequence from positions −739 to +359 (human GRCh37/hg19 chr6 391024-392121) was synthesized by GeneArt (Invitrogen), and the promoter fragment was amplified from the supplied vector (pMK-RQ) using primers to introduce XhoI restriction sites at each end (forward primer 5′-GTCTCGAGATTACAGGCTTGAGCCACA-3′ (underlining indicates enzyme sites) and reverse primer 5′-GACTCGAGCTGGACTCGGAGCTGAGG-3′). The promoter was then cloned into the XhoI site of pGL3 basic (Promega) to generate the pGL3IRF4 promoter construct. *IRF4* enhancer 1 (E1) (chr6 377854-379089) was amplified from genomic DNA using primers to introduce NheI and XhoI sites (forward primer 5′-GAGCTAGCATCGCTTGAGGTTGCAGTG-3′ and reverse primer 5′-GTCTCGAGTGAAGCAGGCACTGTGATTC-3′). The XhoI site was end filled using the Klenow fragment of DNA polymerase I, and the E1 fragment was cloned upstream of the promoter into the NheI and SmaI sites of the pGL3 IRF4 promoter construct. E2 (chr6 365659-366654) was amplified by PCR using primers designed to introduce SacI and NheI sites (forward primer 5′-GAGAGCTCAGCCATCTCCATCATCTGGT-3′ and reverse primer 5′-GAGCTAGCATGTGGAACGCTGGTCC-3′) and cloned upstream of E1 into the SacI and NheI sites of the pGL3IRF4 promoter E1 construct.

### Luciferase reporter assays.

DG75 cell lines were electroporated with plasmid DNA at 260 V and 950 μF (Gene Pulser II; Bio-Rad) using 0.4-cm cuvettes, and luciferase assays were carried out as described previously, with some modifications (49). Briefly, DG75 cells were diluted 1:2 into fresh medium 24 h prior to electroporation. For transfection, cells were pelleted, and the conditioned medium was reserved for later use. Cells were then resuspended in serum-free medium to a density of 2 × 10^7^ cells/ml. A total of 500 μl of the cell suspension was premixed with DNA and then added to the cuvette and immediately electroporated. Transfected cells were then transferred to 10 ml of prewarmed conditioned medium and cultured for 48 h in a humidified incubator at 37°C with 5% CO_2_.

Cells were transfected with 2 μg of the pGL3 luciferase reporter plasmids and 0.5 μg pRL-TK (Promega) as a transfection control, where indicated. Transfection reaction mixtures also included 10 or 20 μg of the EBNA2-expressing plasmid (pSG5 EBNA2), 5 or 10 μg of the IRF4-expressing plasmid (pCMV6XL5-IRF4; Cambridge Biosciences), or the empty vector control. One-tenth of each transfection mixture was processed for Western blotting to analyze EBNA2, IRF4, and actin protein expression levels. The remaining cells were lysed, and firefly and Renilla luciferase activities were measured using the dual-luciferase assay (Promega) and a Glowmax multi detection system (Promega). For transfections where IRF4 was expressed, firefly luciferase signals were normalized to actin expression.

### CRISPR.

CRISPR guides were designed to excise *miR-155HG* enhancer 2 from the B cell genome in the IB4 LCL by targeting genomic regions located 5′ and 3′ of the enhancer. Guides were selected that had on-target and off-target scores that were above 60% (26873822 [CTATCCTTAACAGAACACCC] and 26875376 [TTTAACTAGAACCTTAGACA]) and then ordered as TrueGuide modified synthetic sgRNAs from GeneArt (Invitrogen). IB4 cells were diluted 1:2 into fresh medium 24 h prior to transfection. A total of 1 × 10^6^ cells were then washed in phosphate-buffered saline (PBS), pelleted, and resuspended in 25 μl of resuspension buffer R (Invitrogen). The guide RNA-Cas9 mix was prepared by adding 7.5 pmol of GeneArt TrueCut Cas9 protein V2 (Invitrogen) and 7.5 pmol of sgRNAs to 5 μl of resuspension buffer R and incubating the mixture at room temperature for 10 min. Five microliters of the cell suspension (2 × 10^5^ cells) was then mixed with 7 μl of the Cas9-sgRNA complex. Ten microliters of the cell Cas9-sgRNA mix was then electroporated using the Neon transfection system (Invitrogen) at 1,700 V for 20 ms with 1 pulse. Transfections were carried out in duplicate, and electroporated cells were immediately transferred to two separate wells of a 24-well plate containing 0.5 ml of prewarmed growth medium. Cells were kept in a humidified incubator at 37°C with 5% CO_2_ for 72 h. Cells were then sequentially diluted over a period of 2 weeks and subjected to limited dilution in 96-well plates to obtain single-cell clones. Cell line clones were screened by PCR for genomic deletion using the Phire tissue direct PCR master mix kit (Thermo Scientific). The forward primer (5′-AAATTCCGTGGCTAGCTCCA-3′) hybridized to a region 5′ of enhancer 2, and the reverse primer targeted a region 3′ of enhancer 2 (5′-CTGCTAAGGGAATGTTGAACAAA-3′). Deletions were confirmed by DNA sequencing of the PCR product generated using the forward PCR primer.

### SDS-PAGE and Western blotting.

SDS-PAGE and Western blotting were carried out as described previously (49, 50), using the anti-EBNA2 monoclonal antibody PE2 (gift from M. Rowe), anti-actin at a 1:5,000 dilution (catalog number A-2066; Sigma), and anti-IRF4 at a 1:2,000 dilution (catalog number sc6059; Santa Cruz). Western blot visualization and signal quantification were carried out using a Li-COR imager.

### ChIP-QPCR.

ChIP-QPCR for RBPJ was carried out as described previously (23). *miR-155HG* locus primers were located in the *miR-155HG* promoter (forward primer 5′-AGCTGTAGGTTCCAAGAACAGG-3′ and reverse primer 5′-GACTCATAACCGACCAGGCG-3′), *miR-155HG* enhancer 1 (forward primer 5′-ACCTGTTGACTTGCCTAGAGAC-3′ and reverse primer 5′-TTCTGGTCTGTCTTCGCCAT-3′), a “trough” region between *miR-155HG* enhancer 1 and enhancer 2 (forward primer 5′-TATTCAGCTATTCCAGGAGGCAG-3′ and reverse primer 5′-GTGACATTATCTGCACAGCGAG-3′), and *miR-155HG* enhancer 2 (forward primer 5′-CCTAGTCTCTCTTCTCCATGAGC-3′ and reverse primer 5′-AGTTGATTCCTGTGGACCATGA-3′). *IRF4* locus primers were located in the *IRF4* promoter (forward primer 5′-TCCGTTACACGCTCTGCAA-3′ and reverse primer 5′-CCTCAGGAGGCCAGTCAATC-3′), a trough region between the *IRF4* promoter and enhancer 1 (forward primer 5′-TGTGACAAGTGACGGTATGCT-3′ and reverse primer 5′-TTGTAACAGCGCCTAATGTTGG-3′), *IRF4* enhancer 1 (forward primer 5′-TTACCACCTGGGTACCTGTCT-3′ and reverse primer 5′-ACAGTAGCATGCAGCACTCTC-3′), and *IRF4* enhancer 2 (forward primer 5′-AGTGAGACGTGTGTCAGAGG-3′ and reverse primer 5′-AAGCAGGCACTGTGATTCCA-3′).

### RT-QPCR.

Total RNA was extracted using Tri reagent (Sigma), and RNA samples were then purified using the RNeasy kit (Qiagen). RNA concentrations were determined using a NanoDrop 2000 instrument (Thermo Scientific), and 1 μg was used to prepare cDNA using the ImProm II reverse transcription kit with random primers (Promega). Quantitative PCR was performed in duplicate using the standard curve absolute quantification method on an Applied Biosystems 7500 real-time PCR machine as described previously (22), using previously reported QPCR primers for *miR-155HG* (BIC) (28) (forward primer 5′-ACCAGAGACCTTACCTGTCACCTT-3′ and reverse primer 5′-GGCATAAAGAATTTAAACCACAGATTT-3′) and glyceraldehyde-3-phosphate dehydrogenase (GAPDH) (forward primer 5′-TCAAGATCATCAGCAATGCC-3′ and reverse primer 5′-CATGAGTCCTTCCACGATACC-3′).

### Capture Hi-C.

Previously described capture Hi-C data from GM12878 and CD34^+^ cells were examined for interactions that were captured using baits comprising a 13,140-bp HindIII fragment encompassing the *miR-155HG* promoter (GRCh38/hg19 chr21:26926437-26939577) and a 2,478-bp HindIII fragment that encompasses the miR-155 genomic sequence in exon 3 (GRCh38/hg19 chr21:26945874-26948352).
