# Correction for Wood et al., “Enhancer Control of MicroRNA miR-155 Expression in Epstein-Barr Virus-Infected B Cells”

**DOI:** 10.1128/JVI.01893-18

**Published:** 2019-01-17

**Authors:** C. David Wood, Thomas Carvell, Andrea Gunnell, Opeoluwa O. Ojeniyi, Cameron Osborne, Michelle J. West

**Affiliations:** a School of Life Sciences, University of Sussex, Falmer, Brighton, United Kingdom; b Department of Medical and Molecular Genetics, King's College London School of Medicine, Guy's Hospital, London, United Kingdom

## AUTHOR CORRECTION

Volume 92, no. 19, e00716-18, 2018, https://doi.org/10.1128/JVI.00716-18. Page 5, Fig. 2D: Due to errors in final figure assembly, the actin loading control blots were placed in the incorrect positions under the left and right sections. The right-hand blot should be on the left, and the left-hand blot should be on the right.

Figure 2D should appear as shown below.

**Figure:**
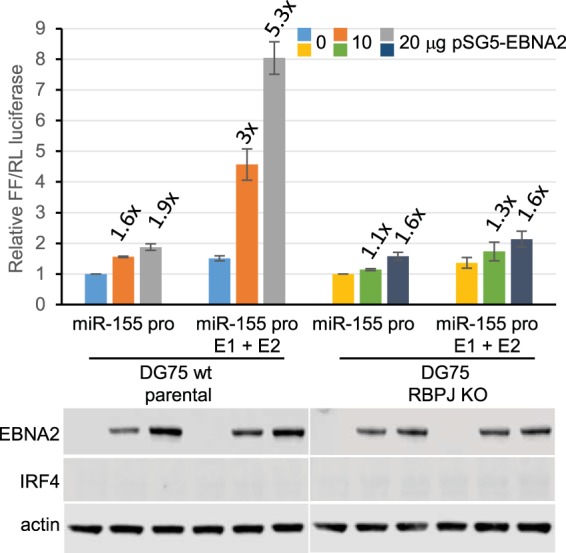


Page 7, Fig. 3: The actin loading control panels were incorrectly sized and cropped during final figure assembly. The left-hand blot has had 9 lanes compressed to fit under 8 lanes of the blots above in error. This resulted in the right-hand blot having an additional lane that should not have been in the figure. These errors arose due to differences in the way the samples were split over two gels for the IRF4 and actin Western blots (which were performed on the same samples analyzed on parallel gels).

Figure 3 should appear as shown below.

**Figure:**
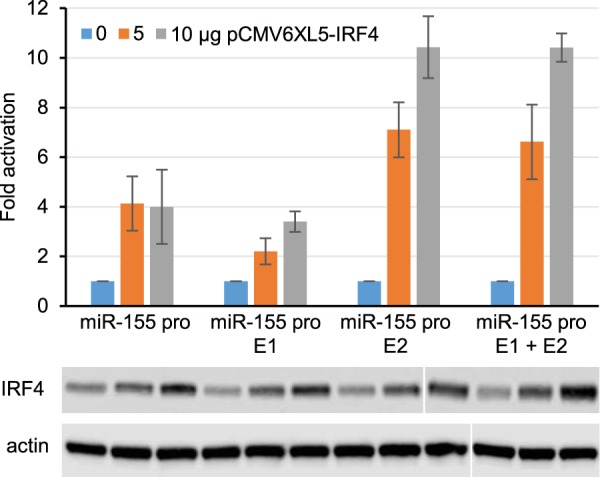


These errors do not affect the conclusions of the paper.

